# The use of milk Fourier transform mid-infrared spectra and milk yield to estimate heat production as a measure of efficiency of dairy cows

**DOI:** 10.1186/s40104-020-00455-0

**Published:** 2020-05-07

**Authors:** Sadjad Danesh Mesgaran, Anja Eggert, Peter Höckels, Michael Derno, Björn Kuhla

**Affiliations:** 1 Institute of Nutritional Physiology “Oskar Kellner,” Leibniz Institute for Farm Animal Biology (FBN), Wilhelm-Stahl-Allee 2, 18196 Dummerstorf, Germany; 2 Institute of Genetics and Biometry, Leibniz Institute for Farm Anih8mal Biology (FBN), Wilhelm-Stahl-Allee 2, 18196 Dummerstorf, Germany; 3 IfM GmbH & Co. KG - Institut für Milchuntersuchung (Milk Testing Services North Rhine-Westphalia), Bischofstraße 85, 47809 Krefeld, Germany

**Keywords:** Dairy cattle, Heat production, Milk spectra, Partial least square regression, Respiration chamber

## Abstract

**Background:**

Transformation of feed energy ingested by ruminants into milk is accompanied by energy losses via fecal and urine excretions, fermentation gases and heat. Heat production may differ among dairy cows despite comparable milk yield and body weight. Therefore, heat production can be considered an indicator of metabolic efficiency and directly measured in respiration chambers. The latter is an accurate but time-consuming technique. In contrast, milk Fourier transform mid-infrared (FTIR) spectroscopy is an inexpensive high-throughput method and used to estimate different physiological traits in cows. Thus, this study aimed to develop a heat production prediction model using heat production measurements in respiration chambers, milk FTIR spectra and milk yield measurements from dairy cows.

**Methods:**

Heat production was computed based on the animal’s consumed oxygen, and produced carbon dioxide and methane in respiration chambers. Heat production data included 168 24-h-observations from 64 German Holstein and 20 dual-purpose Simmental cows. Animals were milked twice daily at 07:00 and 16:30 h in the respiration chambers. Milk yield was determined to predict heat production using a linear regression. Milk samples were collected from each milking and FTIR spectra were obtained with MilkoScan FT 6000. The average or milk yield-weighted average of the absorption spectra from the morning and afternoon milking were calculated to obtain a computed spectrum. A total of 288 wavenumbers per spectrum and the corresponding milk yield were used to develop the heat production model using partial least squares (PLS) regression.

**Results:**

Measured heat production of studied animals ranged between 712 and 1470 kJ/kg BW^0.75^. The coefficient of determination for the linear regression between milk yield and heat production was 0.46, whereas it was 0.23 for the FTIR spectra-based PLS model. The PLS prediction model using weighted average spectra and milk yield resulted in a cross-validation variance of 57% and a root mean square error of prediction of 86.5 kJ/kg BW^0.75^. The ratio of performance to deviation (RPD) was 1.56.

**Conclusion:**

The PLS model using weighted average FTIR spectra and milk yield has higher potential to predict heat production of dairy cows than models applying FTIR spectra or milk yield only.

## Background

The conversion of feed energy (gross energy) ingested by ruminants into human-edible food energy such as milk and meat is accompanied by energy losses in form of fecal and urine excretions, fermentation gases (e.g. methane) and heat. For dairy cows, the proportion of feed converted to milk is defined as feed conversion ratio (FCR) which is only one of numerous measures of efficiency. High-feed efficient cows were described to have a better organic matter digestibility thus providing more digestible energy relative to less efficient counterparts [1]. Some fraction of the digestible energy is converted into methane energy, and high feed conversion efficient cows were found to loose less methane energy per kg dry matter intake (DMI) than less efficient counterparts [2]. The remaining energy fraction, called metabolizable energy (ME) is used by the animal for maintenance and production purposes. Recent findings suggested that the use of ME and thus the post-absorptive metabolism exerts a greater impact on feed efficiency of dairy cows than the digestive performance [3]. First experimental evidence was provided in a study by Derno et al. [4], demonstrating that high compared to low metabolic efficient dairy cows have a lower ME intake, while milk yield did not differ between efficiency groups. During the utilization of ME, heat is produced, whose level for an adult dairy cow is primarily determined by energy requirements for maintenance of the animal and energy secreted in milk. As maintenance energy is not different between cows differing in feed efficiency, it seems that high metabolic efficient cows loose less heat during milk production [4]. Besides, high genetic merit cows partition more ME into milk throughout different lactation stages in comparison with their low genetic merit counterparts [5]. As a noticeable difference of heat production (HP) between individual cows has been described [6], it seems likely that, at a comparable level of milk yield, the level of HP can serve as an indicator of metabolic efficiency.

The measurement of HP from ruminants can be performed by indirect calorimetry. To this end, the daily amount of oxygen (O_2_) consumption, carbon dioxide (CO_2_) production and methane (CH_4_) emission along with urinary nitrogen (N_u_) excretion is measured in respiration chambers (RC) [7]. In spite of the accuracy of measurement, RC are costly and working with them is time-consuming and laborious. Therefore, the development of other time and cost-beneficial approaches to estimate HP of dairy cows is desirable.

Infrared spectroscopy is a high-throughput method and its broad application in livestock sector became more popular in the last few years. For the analysis of milk components e.g., fat, protein, lactose and urea, Fourier transform mid-infrared (FTIR) spectrometry has been developed and refined, and nowadays is frequently used routinely to control milk quality and energy [8]. Besides, FTIR spectra can be used to analyze milk fatty acid concentration and composition, which in turn have been exploited as a proxy for estimating different physiological traits such as energy balance [9] and CH_4_ emission [10]. Furthermore, FTIR spectra reflecting all IR-absorbing milk constitute were used as indicators of cow’s health, energy status, feed intake, and CH4 emission [11–14]. To our knowledge, no study has been conducted yet to predict HP of dairy cows from milk FTIR spectroscopy. Hence, the objective of the present study was to examine if FTIR spectra as a measure of milk energy concentration together with milk yield as a measure of quantity can be used to estimate HP of dairy cows as determined by RC experiments.

## Methods

### Data collection

The obtained data for this study originated from four different experimental projects, which have been conducted at the Leibniz Institute for Farm Animal Biology (FBN) in Dummerstorf, Germany (Additional file 1; Table S1) between 2014 and 2019 (unpublished data).

Animal handling during all projects was carried out according to the instructions for the use of animals as experimental subjects of the State Government in Mecklenburg-Western Pomerania. Experimental protocols were confirmed by the local animal ethics committee (Landesamt für Landwirtschaft, Lebensmittelsicherheit und Fischerei Mecklenburg-Vorpommern). Animals were transferred into RC with a gas recovery rate of 99.9% ± 0.96% and a light cycle of 06:00 to 19:00 h at 15 °C [15]. After 14 h gas equilibration, the gas exchange measurements started at 07:00 h and lasted for two consecutive days from which the daily mean was calculated. Cows had free access to water and were fed twice per day at 07:00 and 15:30 h for ad libitum intake. Feed was offered as total mixed ration during the measurement period and was sampled for assessing dry matter and nutrient composition (Table 1). Feed analysis was conducted by Landwirtschaftliche Untersuchungs- und Forschungsanstalt (LUFA) in Rostock, Germany.

**Table 1:** Ingredients, nutrient composition and energy content of the diets fed to the animals during the respiration chamber experiments

Item	Minimum	Maximum	Mean	Median	SD
Ingredients, g/kg of DM					
Grass silage	500	915	692	680	81
Corn silage	238.0	452.5	360.0	375.0	93.3
Barley straw	42	423	214	259	93
Concentrate	4.6	27.9	17.7	17.8	3.4
Nutrient composition					
DM, g/kg	353	941	529	388	235
NDF^a^, g/kg of DM	275	497	377	377	51
ADF^b^, g/kg of DM	146	254	197	197	23
Crude protein, g/kg of DM	122	171	152	155	13
Crude fat, g/kg of DM	24	33	30	30	2
Ash, g/kg of DM	38	135	71	69	19
ME^c^, MJ/kg of DM	8.5	11.6	10.3	10.5	0.7

^a^Neutral detergent fiber

^b^Acid detergent fiber

^c^Metabolizable energy

The airflow through the chamber was roughly 30 m^3^ per hour and measured by a differential-pressure type V cone flow meter (McCrometer, Hemet, CA). Concentrations of CO_2_ and CH_4_ in the chamber were analyzed by infrared-absorption and the concentration of O_2_ was analyzed paramagnetically (SIDOR SICK AG, Waldkirch, Germany) every 6 min. The body weight (BW) of the animals was determined directly before and after the stay in the RC to calculate the mean metabolic body weight (mBW):

$$ mBW={BW}^{0.75} $$

Heat production was computed using the Brouwer [7] equation based on the measurements of O_2_ consumption and CO_2_ and CH_4_ production and normalized to mBW:

$$ HP/ mBW\left( kJ/{kg}^{0.75}\right)=\left[16.18\ {V}_{O2}(L)+5.02{V}_{CO2}(L)-2.17{V}_{CH4}(L)-5.99{N}_u(g)\right]/ mBW\left({kg}^{0.75}\right) $$

The N_u_ excretion was estimated to be 150 g/d based on comparable diet compositions and feed intake levels [16], thereby accepting an error of 1% in HP. The HP and mBW data were acquired from 168 measurements including multiple observations from 64 German Holstein and 20 Fleckvieh (dual purpose Simmental) cows. As repeated measurements on the same cow were performed at different stages of lactation (e.g. early, mid and late), diets or lactation numbers, each observation was considered independent [17]. Cows were milked two times per day at 07:00 and 16:30 h in the RC. Milk yield was recorded, and a representative subsample of each milking (*n* = 336) was collected and analyzed by the Milk Testing Services North Rhine-Westphalia by a Fourier Transform InfraRed spectrophotometer MilkoScan FT 6000 (Foss, Hillerød, Denmark) following the International Standard Organization 9622 [18]. Spectra were transformed to absorbance by the following equation: absorbance = log (1/transmittance). Spectral outliers were detected using a robust Mahalanobis distance test as introduced by Todorov and Filzmoser [19]. This is a multivariate approach using all generated latent variables (PLS components) at once. Hardly any spectral outlier was detected based on the outcome of the test. Each FTIR milk spectrum encompassed 1060 infrared frequencies (wavenumbers), in a range from 925 to 5008 cm^− 1^. From these, selected wavenumbers (*n* = 288) including three spectral regions, 968–1577, 1720–1808, and 2564–2965 cm^− 1^. The spectra regions were shown to contain crucial information on different milk constitutes [20]. The irrelevant FTIR spectra points, i.e. high water absorptions and O–H bending [21], were removed prior further analysis.

### Heat production prediction model development and validation

To analyze the predictive ability of milk yield alone on HP, we first applied a univariate linear model using the “lm” function in R [22], referred to as L1 model:

$$ {Y}_i=\mu +{\beta}_i Milk\ yield+{\varepsilon}_i $$

,where Y_*i*_ is the HP of animal as response variable, ß_*i*_ is the regression coefficient, and ε_*i*_ is the random residual. Secondly, the FTIR spectra obtained from the morning and afternoon milking were averaged to estimate HP without considering milk yield (M1). In a third approach, the average of two absorption spectra from the evening and morning milking was calculated and subsequently multiplied by the corresponding daily milk yield (M2). Finally, the milk yield-weighted average of the spectra was applied to, in which absorption spectra from morning and afternoon milking were multiplied with the respective milk yield of the morning and afternoon milking (M3). Partial least squares (PLS) regression is widely accepted as the preferred method to analyze the potential relationship between the predictor, i.e. spectral data, and the related physiological outcome [23]. Therefore, the “pls” package [24] implemented in R [22] was employed for M1, M2 and M3 in order to predict HP of cows from the computed milk FTIR spectra and corresponding milk yield. The SIMPLS algorithm in PLS analysis was utilized in order to generate the Latent variables. The models were generated with or without pre-processing of the computed spectra, i.e. any mathematical pre-treatments such as first and second derivatives, of the spectral data. We could not detect any significant improvement in either of the models after spectra processing, therefore the mean and weighted average raw spectra were used in this study. The evaluation of the models was carried out by computing the mean squared error of prediction (MSEP) based on the formula:

$$ MSEP\kern0.5em =\kern0.5em {\sum}_{i=1}^n{\left({O}_i\kern0.5em -\kern0.5em {P}_i\right)}^2\kern0.5em /\kern0.5em n, $$

where *n* represents the total number of observations, and *O*_*i*_ and *P*_*i*_ depict the observed and predicted HP, respectively. The square root of the MSEP (RMSEP) indicates the overall error of the prediction. We carried out a random cross validation in order to observe the performance of the developed prediction models with 10 splits and 10 iterations and the outcome was shown as root mean squared error of cross validation (RMSECV) and the coefficient of determination of cross validation (R^2^CV). This approach allows the observations to be utilized for both calibration and validation, hence each observation used for validation exactly once. Both RMSECV and R^2^CV values were the average of a 10-fold cross validation [17]. Besides, an external 4-way cross-validation was conducted where the data set was split randomly into quarters and the PLS was run 4 times, each time one quarter of data restored for validating the model calibrated on the remaining quarters of the data. The root mean squared error (RMSEV) and R^2^ of external cross-validation (R^2^V) values were computed based on the average of 4 iterations of the external cross-validation. The optimal number of Latent variables (i.e., PLS components) for the prediction model was chosen based on visual observation of the cross-validation RMSE plot against the PLS factors and determining the lowest RMSE.

The concordance correlation coefficient (CCC) was computed using the “epiR” package implemented in R [22]. The CCC analysis is the combination of both precision and accuracy to assess the extent of deviation of data from the line of perfect concordance [25].

### Results and discussion

The descriptive analysis of the animal performance, daily gas measurement and HP (kJ/kg BW^0.75^) is shown in Table 2. The mean (± SD) BW and mBW of the animals during the RC experiments were 692 (± 80) and 134.8 (± 11.7) kg, respectively. The average milk yield of the animals was 25.7 (± 10.3) L/d. The mean and standard deviation of HP normalized to mBW as determined from RC experiments amounted to 1067.2 and 135.9 kJ/kg BW^0.75^, respectively.

**Table 2:** Descriptive analysis of animal performance, gas exchange measurements and computed heat production (*n* = 168)

Item	Minimum	Maximum	Mean	Median	SD
Animal performance					
BW, kg	500	915	692	680	80
mBW^a^, kg^0.75^	105.7	166.4	134.8	133.3	11.7
Lactation number	1.0	10	2.9	3.0	1.6
Days in milk, d	42.0	423.0	213.5	259.0	92.8
DMI, kg/d	4.6	27.9	17.7	17.8	3.4
Milk yield, L/d	5.2	51.4	25.7	24.0	10.3
Gas exchange measurements					
O_2_, L	4686	9238	6786	6740	720
CO_2_, L	4615	9313	7072	7099	908
CH_4_, L	299	792	570	579	81
Heat production, kJ/kg BW^0.75^	712	1469	1067	1068	136

^a^Metabolic bodyweight (mBW = BW^075^)

The three spectral regions retained (968–1577, 1720–1808, and 2564–2965 cm^− 1^) for the prediction model are those, which are typically used for quantifying milk fat, protein as well as lactose contents. This was confirmed by the outcome of the loading value plot (Additional file 2; Fig. S1), indicating bands in the approximate regions around 970–1600, 1700–1800 and 2600–3000 cm^− 1^ to be most important for estimating HP. The wavenumbers around 1175 cm^− 1^ stretching the triacylglycerol ester C–O linkage, C=O stretching (approx. 1750 cm^− 1^), and acyl chain C–H symmetric and asymmetric stretching (2800–3000 cm^− 1^) are generally important to determine milk fat content [26]. The amide I, II and III bands (1200–1700 cm^− 1^) are utilized to assess milk protein content [27]. The bond between carbon atom and hydroxyl group, C–OH (around 1080 cm^− 1^) can be used to evaluate carbohydrates such as lactose [28]. The spectral width indicates the energy density of milk but contains no quantitative information on the energy secretion of an animal. Hence, the retained milk FTIR spectra in this study were multiplied by the corresponding daily milk yield prior to the model development. This procedure has been applied also by others, computing the daily energy secreted in milk by multiplying daily milk yield with the equivalent energy of major milk constitutes [29]. A relation between multiple traits originating from milk constitutes e.g. milk fatty acid compositions and milk-to-protein ratio estimated from FTIR spectral data, and energy balance in dairy cattle were postulated [30, 31]. Based on this knowledge, we expected to find an evident correlation between the selected spectra regions, depicting aforementioned milk components and the level of HP of dairy cows.

The linear model L1 resulted in the coefficient of determination of 0.46. The M1 prediction model, without consideration of milk yield, resulted in a RMSEP value of 99.9 kJ/kg BW^0.75^ and a R^2^ value of 0.23 (Table 3). The cross-validation of the M1 model yielded a RMSECV and R^2^CV value of 86.7 kJ/kg BW^0.75^ and 0.25, respectively. The RMSEV and R^2^CV values of the external validation of M1 were 114.1 kJ/kg BW^0.75^ and 0.18, respectively. The PLS prediction model M2, involving the average of the morning and afternoon spectra as well as milk yield, resulted in a RMSEP and R^2^ of 93.2 kJ/kg BW^0.75^ and 0.52, respectively (Table 3). For the cross-validation of the latter model, the RMSECV and R^2^CV values were 89.4 kJ/kg BW^0.75^ and 0.55, respectively, and for the external validation 84.0 kJ/kg BW^0.75^ and 0.48, respectively. The results for the prediction model M3, considering the weighted average milk spectra and milk yield, were even slightly better with a RMSEP and R^2^ of 91.2 kJ/kg BW^0.75^ and 0.54, respectively (Table 3). The cross-validation of the M3 model resulted in RMSECV and R^2^CV of 86.5 kJ/kg BW^0.75^ and 0.57, respectively. However, the external validation approach of the M3 model showed RMSEV and R^2^ values of 95.5 kJ/kg BW^0.75^ and 0.47, respectively. These results show that the involvement of FTIR spectra improves the prediction accuracy of HP as compared to the L1 model considering milk yield only.

**Table 3:** The statistics of partial least square regression approach for the milk Fourier transform mid-infrared spectrometry-based estimation model for heat production of dairy cows

Trait	Prediction model	Calibration		LV^c^	Cross Validation		External Validation	
		R^2^	RMSEP^b^		R^2^CV	RMSECV^d^	R^2^V	RMSEV^e^
Heat production, kJ/kg BW^0.75^	M1^a^	0.23	99.9	14	0.25	86.7	0.18	114.1
	M2^a^	0.52	93.2	4	0.55	89.4	0.48	84.0
	M3^a^	0.54	91.2	5	0.57	86.5	0.47	95.5

^a^Model M1 was developed using the averaged morning and afternoon spectral data. The prediction model M2 was developed by averaging the morning and afternoon spectral data and subsequent multiplication with daily milk yield. The prediction model M3 was computed by weighted averaging, where each morning or afternoon absorption spectra was multiplied to the respective milk yield

^b^The square root of the mean squared error of prediction

^c^Latent variables; i.e. the partial least square regression components for the prediction model

^d^Root mean squared error of cross validation

^e^Root mean squared error of external validation

**Fig. 1:**
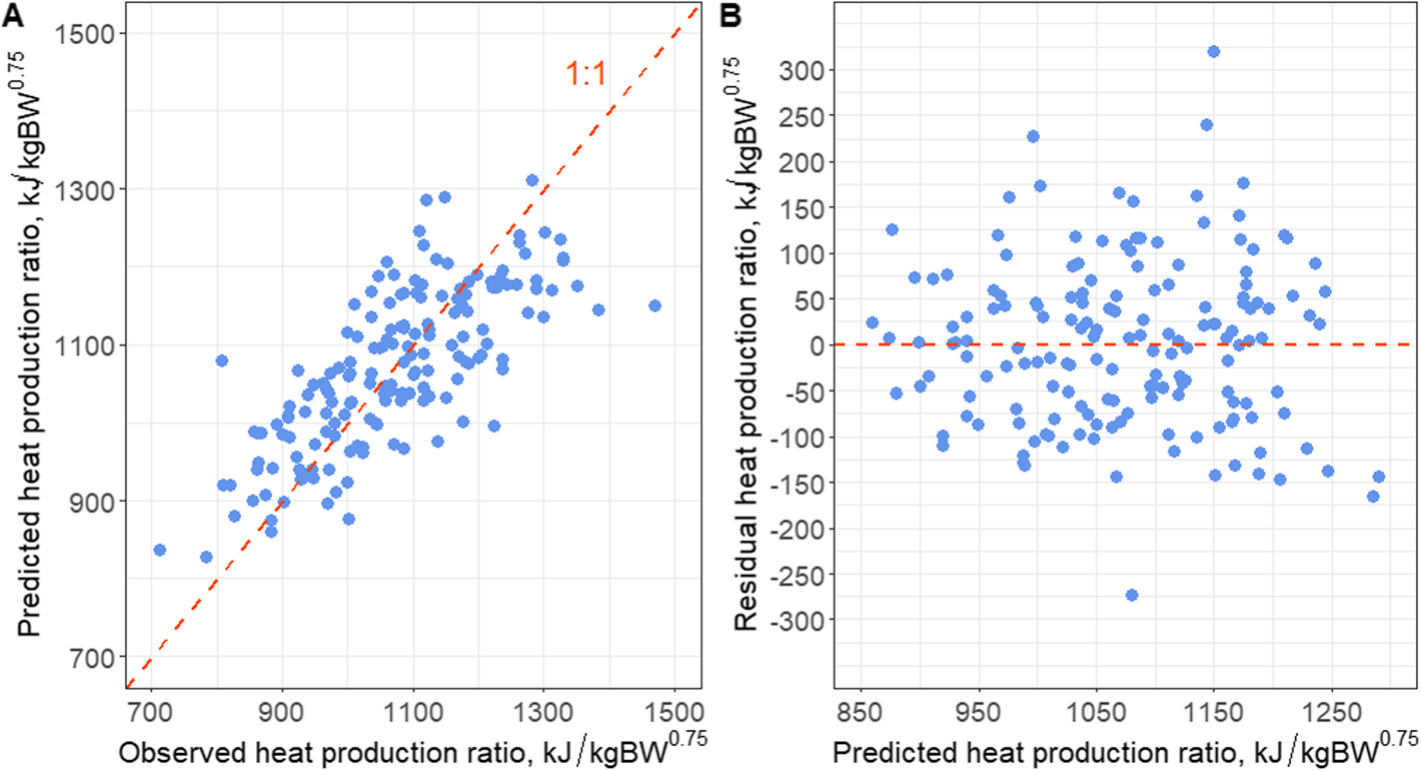
Predicted against observed measurements (**a**) and residual against predicted measurements (**b**) applying the milk Fourier transform mid-infrared spectrometry partial least square (PLS) regression model M2 predicting heat production (HP) of dairy cattle in respiration chamber. Prediction model M2 was developed by averaging the morning and afternoon spectral data and multiply to the milk yield at the day of sampling

**Fig. 2:**
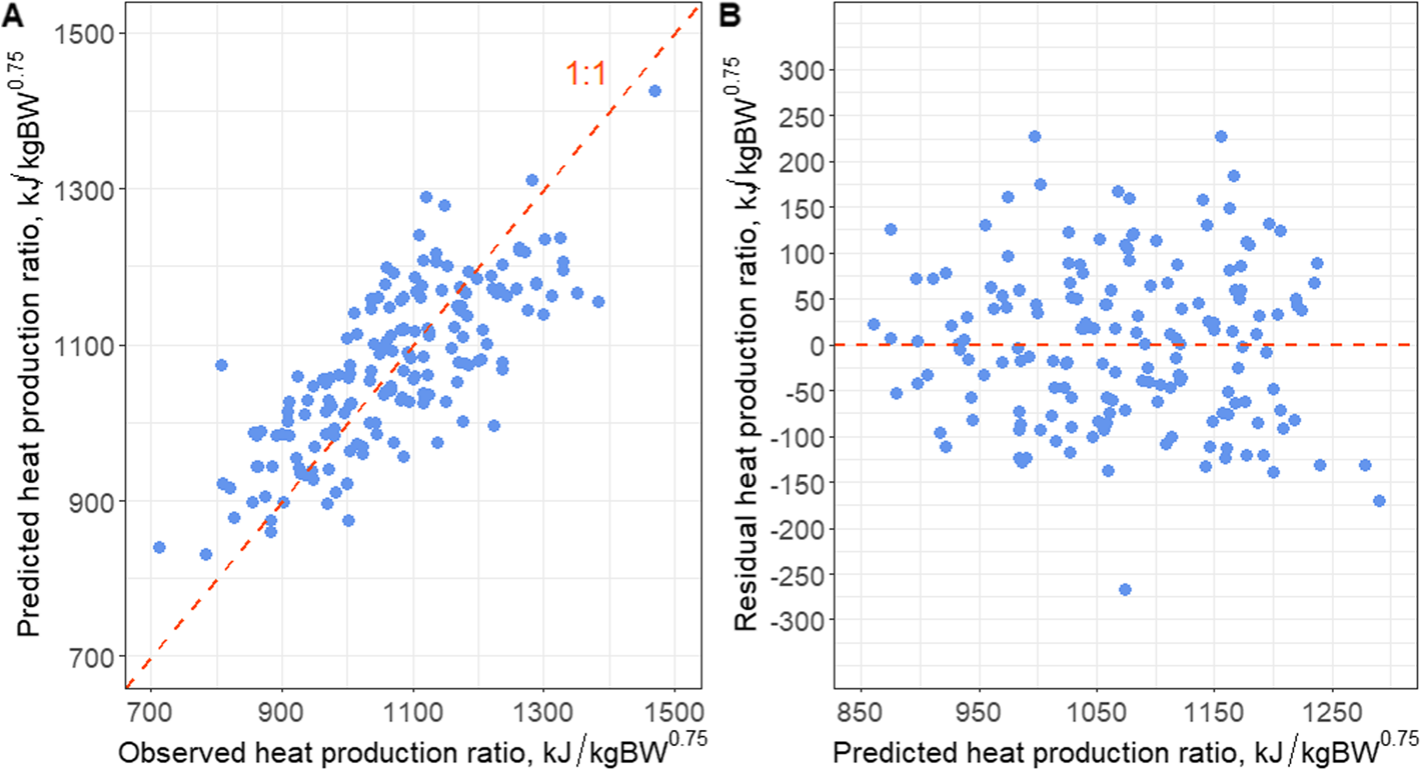
Predicted against observed measurements (**a**) and residual against predicted measurements (**b**) applying the milk Fourier transform mid-infrared spectrometry partial least square (PLS) regression model M3 predicting heat production (HP) of dairy cattle in respiration chamber. Prediction model M3 was computed by weighted averaging the morning and afternoon milk spectral data, in which each morning or afternoon absorption spectra was multiplied to the respective milk yield and subsequently the average spectrum was computed

The predicted against observed and residuals versus predicted HP of the FTIR spectra- and milk yield-based estimation models M2 and M3 are shown in Fig. 1a and b and Fig. 2a and b, respectively. Based on the results obtained it can be concluded that including milk yield into the prediction model improves the accuracy for estimating HP relative to the prediction which is based on FTIR spectra only (M1). McParland et al. stated that adding milk yield to the FTIR spectra-based model increased the prediction accuracy for body energy status in dairy cows [12]. Moreover, Shetty et al. [32] showed that including milk yield improved the overall performance of the CH_4_ estimation model than utilizing FTIR wavenumbers only. These earlier findings agree with our results showing that applying milk yield in the final transformation of the spectra as a factor slightly improved the performance of models than using milk yield as a predictor variable. Furthermore, previous studies indicated that the use of milk yield-weighted average compared to the average of FTIR spectra is more biologically relevant [14, 33]. However, the negligible differences of R^2^ between calibration and validation (< 10%) between M2 and M3 show the fair robustness of both prediction models. The concordance correlation coefficient (CCC) analysis pinpointed a substantial predictive ability of our FTIR-based models (CCC of 0.67 and 0.71 for M2 and M3 models, respectively) [34]. Although both M2 and M3 models showed overall similar performances, M3 indicated a marginally better outcome. Nevertheless, the predicted mid infrared spectra-based model explained only 57% of the variation of dairy cows HP. Previous studies have exploited the PLS approach to develop milk FTIR-based models for estimating other traits such as CH_4_ emission or body energy status, yet clear disparities between model performances can be observed [17, 32, 35–37]. A recent work demonstrated a milk FTIR spectra-based CH_4_ production estimation model using observations obtained from a non-invasive CH_4_ measurement approach using the sniffer method [32]. Despite the efforts from these authors to increase the robustness of the model e.g., by conducting an external validation using independent observations, the prediction accuracy for CH_4_ production from milk FTIR wavenumbers resulted in a R^2^ of validation of 0.13 only [32]. van Gastelen et al. [17] determined the possibility to use milk FTIR as a proxy for CH_4_ emission in dairy cows by generating a prediction PLS model for CH_4_ measurements acquired from RC experiments. Their prediction model showed a R^2^CV of 0.30 for CH_4_ production [17], which is lower than the R^2^CV of our present models, particularly M2 and M3 models. On the other hand, Smith et al. [37] collected over 11,500 records from 1101 Holstein Friesian cows to generate a mid-infrared spectra-based regression model for energy balance of dairy cows. The predictive ability of the model revealed R^2^ of internal and external cross-validation of 0.77 and 0.60, respectively [37], which are higher than the R^2^ values of our models. Vanlierde et al. [36] collated the total number of 584 reference data from CH_4_ measurements executed in RC of different European cattle research centers, including different breeds and feeding managements. The predictive ability of the model for CH_4_ emission indicated R^2^ of calibration and cross-validation of 0.65 and 0.57, respectively [36], which are similar to the R^2^ values of FTIR-based models of this work. The modest R^2^ values obtained from the present estimation models call for a need to enlarge the current data set, but this can only be achieved in future studies. Besides, CH_4_ production is only one component of HP, and the variation of O_2_ consumption and CO_2_ production as further variables of HP certainly contribute to the lower R^2^ as well. Another explanation may be that we considered multiple measurements on the same animal as independent due to changes in diet or physiological state. On the other hand it has been shown that the R^2^ value is improved when repeated CH_4_ emission measurements i.e., recorded during a 7-day measurement period, were incorporated in the prediction model [14, 33]. There are general concerns on applying FTIR spectroscopy as a prediction tool for physiological traits of cows, which cannot be overseen. Van Gastelen and Dijkstra [38] reported on the inability of FTIR spectroscopy to detect various lower abundant milk fatty acids, which indeed are important for the prediction of CH_4_ production from milk fatty acids analyzed by gas-chromatography. The same limitation might be applicable for the HP estimation in the present study, as spectra wavenumbers associated with milk fat content is apparently among the important regions for predicting HP level of dairy cows.

The coefficient of determination greatly relies on the range and variability of the observations. Despite considering observations from experiments containing different dietary compositions and quite a range of DMI, BW and milk yield, the current FTIR-based models seem still not to have covered the high-enough variability to reach a higher rate of HP predicting accuracy. The higher predictive ability of models generated by Dehareng et al. [14] and Vanlierde et al. [33] with R^2^CV of 0.68–0.79 and 0.77, respectively, is likely due to the wide range of CH_4_ measurements, which were between 218–653 g/d and 180–950 g/d, respectively. A recent study combined the CH_4_ production data obtained from RC and the sulphur hexafluoride (SF_6_) tracer technique, thereby enlarging the dataset to 1089 observations from 299 cows, and thus the variability of CH_4_ production (286–546 g/d) [39]. The PLS model from this study indicated better prediction accuracy in comparison with our model (R^2^ of validation of 0.64 vs. 0.57), again underscoring the need of more data sets for improving the HP prediction model in future.

We also determined the applicability of our models by analyzing the ratio of performance to deviation (RPD). The RPD value is based on the relation between the standard error of prediction to the standard deviation of the referenced observation, in which the higher RPD values pinpoints the more suitability of the model for future screening, quality control as well as any other applications [40]. The prediction models developed herein yielded a relatively small RPD value of 1.51 and 1.56 for M2 and M3, respectively, which were, however, higher than the one established by van Gastelen et al. [17] for estimating CH_4_ production (RPD = 1.19). Conversely, the RPD of our models was lower than the FTIR-based model by Dehareng et al., who reported a RPD of 2.19 [14]. Different classifications of model performance have been proposed based on RPD values, in which models with RPD higher than 2.5 or 3.0 are considered as feasible for screening programs [40, 41]. Nonetheless, Williams [40] acknowledged the difficulties to reach RPD of 3.0 or higher due to different factors associated with infrared spectroscopy technique e.g., sample presentation to the instrument and low variance in the sample set. The RPD value obtained in the present study reveals that the applicability of the FTIR-based models for predicting HP is currently not good enough as it does not provide accurate classification from a cohort with small HP differences [17]. It is important to mention that the RPD value also relies on the range of variability of the observations. Hence, the observations incorporated within this work seem to lack sufficient variation range in order to result in a model with higher RPD value. Extending the range of variation e.g., by implementing more different feeding regimens, cattle genotypes, dietary treatments, etc., could potentially increase the RPD value.

## Conclusion

The current work was the first to show the potential use of milk FTIR spectra to predict HP of dairy cows. The outcome of this study, at least at preliminary stages, revealed that milk FTIR spectra together with milk yield can potentially be used to identify dairy cows with different HP and thus feed efficiency levels. Selection for animals that utilize feed more efficiently would be ideal for dairy managers in order to benefit the financial returns. The FTIR-based models were robust with evident predictive ability; however, they can only describe a moderate part of the observed HP variations. The applicability of the prediction models remained relatively poor, which implies the need to enlarge the current data set. Future work exploiting higher number of observations from a wider range of breeds and feeding regimens seems warranted in order to further ameliorate the quality of the model.

## Supplementary information

**Additional file 1:Table S1.** Detailed description of the respiration chamber (RC) experiments used as data resources for developing the FTIR-based heat production estimation model.

**Additional file 2:Figure S1.** The regression coefficients of the partial least squares model for predicting heat production plotted against the wavenumbers (cm^− 1^). The grey shaded area depicts the spectra regions selected for generating the present partial least square models (968–1577, 1720–1808, and 2564–2965 cm^− 1^).

## Data Availability

All data generated or analyzed are available from the corresponding author on request.

## Abbreviations

CCC: Concordance correlation coefficient
CH_4_: Methane
CO_2_: Carbon dioxide
FTIR: Fourier transform mid-infrared
HP: Heat production
mBW: Metabolic body weight
MSEP: Mean squared error of prediction
N_2_: Nitrogen
O_2_: Oxygen
PLS: Partial least square
RC: Respiration chambers
R^2^CV: Coefficient of determination of cross validation
RMSECV: Root mean squared error of cross validation
RPD: Ratio of performance to deviation

## References

[1] Rius AG, Kittelmann S, Macdonald KA, Waghorn GC, Janssen PH, Sikkema E (2012). Nitrogen metabolism and rumen microbial enumeration in lactating cows with divergent residual feed intake fed high-digestibility pasture. J Dairy Sci.

[2] Arndt C, Powell JM, Aguerre MJ, Wattiaux MA (2015). Performance, digestion, nitrogen balance, and emission of manure ammonia, enteric methane, and carbon dioxide in lactating cows fed diets with varying alfalfa silage-to-corn silage ratios. J Dairy Sci.

[3] Potts SB, Boerman JP, Lock AL, Allen MS, VandeHaar MJ (2017). Relationship between residual feed intake and digestibility for lactating Holstein cows fed high and low starch diets. J Dairy Sci.

[4] Derno M, Nurnberg G, Kuhla B (2019). Characterizing the metabotype and its persistency in lactating Holstein cows: an approach toward metabolic efficiency measures. J Dairy Sci.

[5] Bryant JR, Lopez-Villalobos N, Holmes CW, Pitman GD, Brookes IM (2003). Effect of genetic merit on the estimated partitioning of energy towards milk production or liveweight gain by Jersey cows grazing on pasture. Proceedings of the New Zealand Society of Animal Production; Jan.

[6] Yan T, Gordon FJ, Agnew RE, Porter MG, Patterson DC (1997). The metabolisable energy requirement for maintenance and the efficiency of utilisation of metabolisable energy for lactation by dairy cows offered grass silage-based diets. Livest Prod Sci.

[7] Brouwer E (1965). Report of sub-committee on constants and factors. Energy metabolism of farm animals.

[8] Grelet C, Fernández Pierna JA, Dardenne P, Baeten V, Dehareng F (2015). Standardization of milk mid-infrared spectra from a European dairy network. J Dairy Sci.

[9] Dórea JRR, French EA, Armentano LE (2017). Use of milk fatty acids to estimate plasma nonesterified fatty acid concentrations as an indicator of animal energy balance. J Dairy Sci.

[10] Engelke SW, Daş G, Derno M, Tuchscherer A, Berg W, Kuhla B (2018). Milk fatty acids estimated by mid-infrared spectroscopy and milk yield can predict methane emissions in dairy cows. Agron Sustain Dev.

[11] van Knegsel ATM, van der Drift SGA, Horneman M, de Roos APW, Kemp B, Graat EAM (2010). Short communication: ketone body concentration in milk determined by Fourier transform infrared spectroscopy: value for the detection of hyperketonemia in dairy cows. J Dairy Sci.

[12] McParland S, Banos G, Wall E, Coffey MP, Soyeurt H, Veerkamp RF (2011). The use of mid-infrared spectrometry to predict body energy status of Holstein cows1. J Dairy Sci.

[13] McParland S, Lewis E, Kennedy E, Moore SG, McCarthy B, O'Donovan M (2014). Mid-infrared spectrometry of milk as a predictor of energy intake and efficiency in lactating dairy cows. J Dairy Sci.

[14] Dehareng F, Delfosse C, Froidmont E, Soyeurt H, Martin C, Gengler N (2012). Potential use of milk mid-infrared spectra to predict individual methane emission of dairy cows. Animal..

[15] Derno M, Elsner HG, Paetow EA, Scholze H, Schweigel M (2009). Technical note: a new facility for continuous respiration measurements in lactating cows. J Dairy Sci.

[16] Dijkstra J, Oenema O, van Groenigen JW, Spek JW, van Vuuren AM, Bannink A (2013). Diet effects on urine composition of cattle and N2O emissions. Animal..

[17] van Gastelen S, Mollenhorst H, Antunes-Fernandes EC, Hettinga KA, van Burgsteden GG, Dijkstra J (2018). Predicting enteric methane emission of dairy cows with milk Fourier-transform infrared spectra and gas chromatography-based milk fatty acid profiles. J Dairy Sci.

[18] ISO (2013). International Organization for Standardization-Guidelines for the application of midinfrared spectrometry.

[19] Todorov V, Filzmoser P (2009). An object-oriented framework for robust multivariate analysis. J Stat Softw.

[20] Capuano E, van der Veer G, Boerrigter-Eenling R, Elgersma A, Rademaker J, Sterian A (2014). Verification of fresh grass feeding, pasture grazing and organic farming by cows farm milk fatty acid profile. Food Chem.

[21] Belay TK, Dagnachew BS, Kowalski ZM, Adnoy T (2017). An attempt at predicting blood beta-hydroxybutyrate from Fourier-transform mid-infrared spectra of milk using multivariate mixed models in polish dairy cattle. J Dairy Sci.

[22] Development R. Core team. R: a language and environment for statistical computing. Vienna, Austria: R foundation for statistical. Computing. 2019.

[23] Martens H, Næs T, Kowalski BR (1984). Multivariate Calibration. Chemometrics: mathematics and statistics in chemistry.

[24] Mevik B-H, Wehrens R (2007). The pls package: principal component and partial least squares regression in R. J Stat Softw.

[25] Lin LI (1989). A concordance correlation coefficient to evaluate reproducibility. Biometrics..

[26] Yang H, Irudayaraj J (2000). Characterization of semisolid fats and edible oils by Fourier transform infrared photoacoustic spectroscopy. J Am Oil Chem Soc.

[27] Etzion Y, Linker R, Cogan U, Shmulevich I (2004). Determination of protein concentration in raw Milk by mid-infrared Fourier transform infrared/attenuated Total reflectance spectroscopy. J Dairy Sci.

[28] Hashimoto A, Kameoka T (2008). Applications of infrared spectroscopy to biochemical, food, and agricultural processes. Appl Spectrosc Rev.

[29] Gaspardy A, Schwartz Z, Zoldag L, Veresegyhazy T, Fekete S (2004). Changes in daily energy amounts of main milk components (lactose, protein and fat) during the lactation of high-yielding dairy cows. Acta Vet Hung.

[30] Reist M, Erdin D, von Euw D, Tschuemperlin K, Leuenberger H, Chilliard Y (2002). Estimation of energy balance at the individual and herd level using blood and Milk traits in high-yielding dairy cows. J Dairy Sci.

[31] Friggens NC, Ridder C, Lovendahl P (2007). On the use of milk composition measures to predict the energy balance of dairy cows. J Dairy Sci.

[32] Shetty N, Difford G, Lassen J, Løvendahl P, Buitenhuis AJ (2017). Predicting methane emissions of lactating Danish Holstein cows using Fourier transform mid-infrared spectroscopy of milk. J Dairy Sci.

[33] Vanlierde A, Vanrobays ML, Dehareng F, Froidmont E, Soyeurt H, McParland S (2015). Hot topic: innovative lactation-stage-dependent prediction of methane emissions from milk mid-infrared spectra. J Dairy Sci.

[34] Altman DG (1997). Practical statistics for medical research.

[35] Luke TDW, Rochfort S, Wales WJ, Bonfatti V, Marett L, Pryce JE (2019). Metabolic profiling of early-lactation dairy cows using milk mid-infrared spectra. J Dairy Sci.

[36] Vanlierde A, Soyeurt H, Gengler N, Colinet FG, Froidmont E, Kreuzer M (2018). Short communication: Development of an equation for estimating methane emissions of dairy cows from milk Fourier transform mid-infrared spectra by using reference data obtained exclusively from respiration chambers. J Dairy Sci.

[37] Smith SL, Denholm SJ, Coffey MP, Wall E (2019). Energy profiling of dairy cows from routine milk mid-infrared analysis. J Dairy Sci.

[38] van Gastelen S, Dijkstra J (2016). Prediction of methane emission from lactating dairy cows using milk fatty acids and mid-infrared spectroscopy. J Sci Food Agric.

[39] Denninger TM, Dohme-Meier F, Eggerschwiler L, Vanlierde A, Grandl F, Gredler B (2019). Persistence of differences between dairy cows categorized as low or high methane emitters, as estimated from milk mid-infrared spectra and measured by GreenFeed. J Dairy Sci.

[40] Williams P, The RPD (2014). Statistic: a tutorial note. NIR news.

[41] Williams PC, Sobering DC (1993). Comparison of commercial near infrared transmittance and reflectance instruments for analysis of whole grains and seeds. J Near Infrared Spectrosc.

