# Supplementary material for: The use of milk Fourier transform mid-infrared spectra and milk yield to estimate heat production as a measure of efficiency of dairy cows
Source: J Anim Sci Biotechnol. 2020 May 7;11:43. doi: 10.1186/s40104-020-00455-0 (PMC7204237; doi:10.1186/s40104-020-00455-0)
Supplement: Supplementary file 1 — Additional file 1:Table S1. Detailed description of the respiration chamber (RC) experiments used as data resources for developing the FTIR-based heat production estimation model. [file 40104_2020_455_MOESM1_ESM.docx]

Table S1. Detailed description of the respiration chamber (RC) experiments used as data resources for developing the FTIR-based heat production estimation model.

| RC experiment | No. of animal | Breed | Lactation stage | Dietary characteristics^a^ |
| --- | --- | --- | --- | --- |
| 1 | 38 | German Holstein | mid and late | Two isoenergetic diets with different energy (NEL^b^) levels (6.5 vs. 6.1 MJ/kg of DM from roughage) fed over two consecutive lactations. |
| 2 | 20 | German Holstein | late | Two diets containing low (14.7%) and high (16.7%) CP^c^. Low CP: 32% concentrate, 27% grass silage and 41% corn silage. High CP: 36% concentrate, 31% grass silage and 33% corn silage. |
| 3 | 20 | Fleckvieh | late | Two diets (63% roughage, 37% concentrate) with either only grass or corn silage as roughage. |
| 4 | 6 | German Holstein | early | One diet containing 30% concentrate, 30% grass silage and 40% corn silage. |

^a^DM basis

^b^Net energy for lactation

^c^Crude Protein
