# Supplementary figures and images for: The use of milk Fourier transform mid-infrared spectra and milk yield to estimate heat production as a measure of efficiency of dairy cows
Source: J Anim Sci Biotechnol. 2020 May 7;11:43. doi: 10.1186/s40104-020-00455-0 (PMC7204237; doi:10.1186/s40104-020-00455-0)

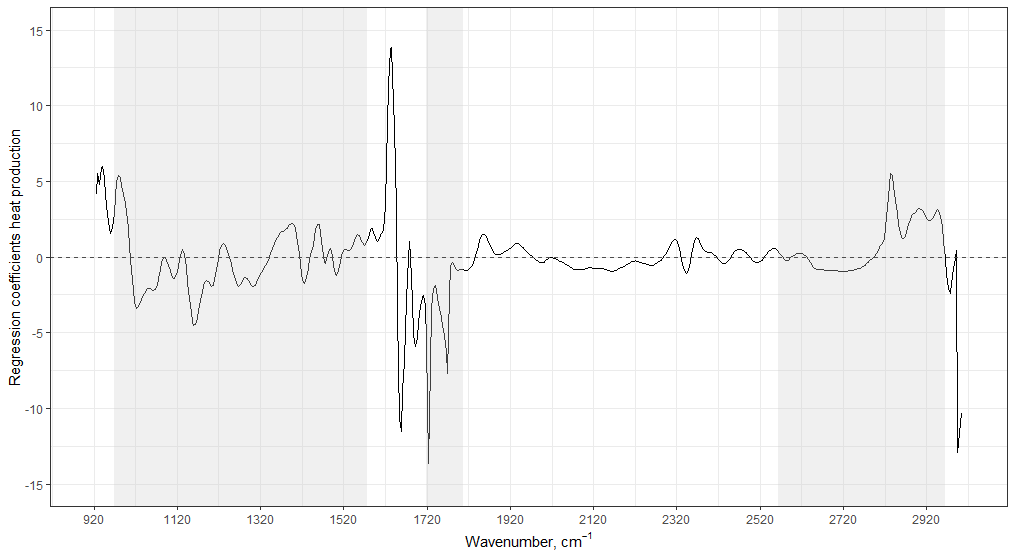

Supplement: Supplementary file 2 — Additional file 2:Figure S1. The regression coefficients of the partial least squares model for predicting heat production plotted against the wavenumbers (cm− 1). The grey shaded area depicts the spectra regions selected for generating the present partial least square models (968–1577, 1720–1808, and 2564–2965 cm− 1). [file 40104_2020_455_MOESM2_ESM.png]
